# Evaluation of Silver Nanoparticle Toxicity in Skin *in Vivo* and Keratinocytes *in Vitro*

**DOI:** 10.1289/ehp.0901398

**Published:** 2009-10-23

**Authors:** Meghan E. Samberg, Steven J. Oldenburg, Nancy A. Monteiro-Riviere

**Affiliations:** 1 Center for Chemical Toxicology Research and Pharmacokinetics, North Carolina State University, Raleigh, North Carolina, USA; 2 Joint Department of Biomedical Engineering, University of North Carolina, Chapel Hill, and North Carolina State University, Raleigh, North Carolina, USA; 3 nanoComposix, San Diego, California, USA

**Keywords:** keratinocytes, metal oxides, nanoparticles, nanotechnology, porcine skin, silver, skin penetration, toxicity

## Abstract

**Introduction:**

Products using the antimicrobial properties of silver nanoparticles (Ag-nps) may be found in health and consumer products that routinely contact skin.

**Objectives:**

This study was designed to assess the potential cytotoxicity of Ag-nps in human epidermal keratinocytes (HEKs) and their inflammatory and penetrating potential into porcine skin *in vivo*.

**Materials and Methods:**

We used eight different Ag-nps in this study [unwashed/uncoated (20, 50, and 80 nm particle diameter), washed/uncoated (20, 50, and 80 nm), and carbon-coated (25 and 35 nm)]. Skin was dosed topically for 14 consecutive days. HEK viability was assessed by MTT, alamarBlue (aB), and CellTiter 96 AQueous One (96AQ). Release of the proinflammatory mediators interleukin (IL)-1β, IL-6, IL-8, IL-10, and tumor necrosis factor-α (TNF-α) were measured.

**Results:**

The effect of the unwashed Ag-nps on HEK viability after a 24-hr exposure indicated a significant dose-dependent decrease (*p* < 0.05) at 0.34 μg/mL with aB and 96AQ and at 1.7 μg/mL with MTT. However, both the washed Ag-nps and carbon-coated Ag-nps showed no significant decrease in viability at any concentration assessed by any of the three assays. For each of the unwashed Ag-nps, we noted a significant increase (*p* < 0.05) in IL-1β, IL-6, IL-8, and TNF-α concentrations. We observed localization of all Ag-nps in cytoplasmic vacuoles of HEKs. Macroscopic observations showed no gross irritation in porcine skin, whereas microscopic and ultrastructural observations showed areas of focal inflammation and localization of Ag-nps on the surface and in the upper stratum corneum layers of the skin.

**Conclusion:**

This study provides a better understanding Ag-nps safety *in vitro* as well as *in vivo* and a basis for occupational and risk assessment. Ag-nps are nontoxic when dosed in washed Ag-nps solutions or carbon coated.

Historically, silver (Ag) compounds have been used in numerous fields to prevent microbial growth. Like many nonessential heavy metals, Ag is a natural biocide, but compared with titanium, zinc, and copper, Ag nanoparticles (Ag-nps) show the highest antimicrobial efficacy against bacteria, viruses, and other eukaryotic microorganisms (Gong et al. 2007). The Phoenicians coated milk bottles with Ag to inhibit bacterial growth; doctors have administered drops of Ag nitrate solutions to newborn babies to prevent neonatal conjunctivitis (Crede 1881); and Ag sulfadiazine creams have long been considered the standard of care for the prevention of widespread bacterial growth on burn patients’ denuded skin (Moyer et al. 1965). Both dietary supplements and homemade varieties of Ag colloids have been sold for decades as a “cure-all” for such diseases as tuberculosis, syphilis, scarlet fever, shingles, herpes, pneumonia, and arthritis (National Center for Complementary and Alternative Medicine 2009). Furthermore, advances in nanotechnology have facilitated the increase of Ag-containing merchandise available to the public, making Ag the most used nanomaterial of all manufacturer-identified products in the world (Project on Emerging Nanotechnologies 2009). Products such as room deodorizing sprays, acne creams, clothing that prevents body odor, baby wipes, and pacifiers all exploit the natural antimicrobial activity of Ag (Project on Emerging Nanotechnologies 2009). In a study investigating the release of Ag-nps from commercially available sock fabric, Benn and Westerhoff (2008) showed that socks could contain up to 1,360 μg Ag/g per sock and could release as much as 1.3 μg/mL of Ag into distilled water.

Ingestion of Ag can cause argyria, the benign condition characterized by the bluish-graying of the skin that occurs through the preferential deposition of Ag in the basal lamina of soft tissues such as the skin, liver, and spleen (Fung and Bowen 1996) and blood vessels, gastrointestinal tract, liver, and kidney (Danscher 1980). Although argyria is most commonly reported clinically after excessive Ag ingestion, Ag deposition has been seen after treatment of burned skin with Ag sulfadiazine (Lee and Lee 1994; Marshall and Schneider 1977; Temple and Farooqi 1985). In response to argyria, but not to Ag toxicity, the National Institute for Occupational Safety and Health (2003) set a daily exposure limit for all forms of Ag at 0.01 mg/m^3^, and the U.S. Environmental Protection Agency (2003) established the oral reference dose at 0.005 mg/kg/day.

Studies indicating Ag toxicity exist from as early as 1983, when Rungby and Danscher (1983) showed that Ag salts intraperitoneally administered to rats can accumulate in neurons and in protoplasmic glial cells of the brain and spinal cord. *In vitro* cell line studies have shown decreased mitochondrial function after exposure to Ag-nps in murine neuroblastoma cells (Schrand et al. 2008), hepatic cells (Hussain et al. 2005), germline stem cells (Braydich-Stolle et al. 2005), human skin carcinoma cells (Arora et al. 2008), and human epidermal keratinocytes (HEKs) and fibroblasts (Burd et al. 2007). Although *in vivo* studies have not been performed with Ag-nps, polyvinylpyrrolidone-stabilized Ag-nps with a mean size of 25 nm were shown to penetrate into the upper layers of the epidermis in excised human skin in static diffusion cells (Larese et al. 2009). Other nanomaterials, such as quantum dots (QDs) and fullerenes (Rouse et al. 2006; Ryman-Rasmussen et al. 2006; Zhang and Monteiro-Riviere 2008; Zhang et al. 2008), as well as zinc oxide (Cross et al. 2007; Gamer et al. 2006), are able to penetrate into the stratum corneum; thus, examination of the ability of Ag-nps to penetrate the skin is warranted.

Our objectives in this study were to determine the optimal viability assay for use with Ag-nps in order to assess their toxicity to skin cells, their inflammatory potential, and capacity to penetrate skin.

## Materials and Methods

### Ag-nps

To encompass the variety of manufactured Ag-nps on the market, we used eight different forms of Ag-nps with different sizes and surface conditions (Table 1). All Ag-nps used in this study were supplied by nanoComposix (San Diego, CA): commercially used unwashed and uncoated Ag-nps suspended in deionized water with diameters of 20, 50, and 80 nm (“unwashed”); washed and uncoated Ag-nps suspended in deionized water with diameters of 20, 50, and 80 nm (“washed”); and commercially used dried carbon-coated Ag-nps with diameters of 25 and 35 nm (“carbon-coated”). The sizes of each type of Ag-nps were determined by the manufacturer and confirmed in this study by dynamic light scattering (DLS) and transmission electron microscopy (TEM).

Both the unwashed and washed Ag-nps were synthesized by ammonium hydroxide– catalyzed growth of Ag onto 5-nm gold seed particles. Concentration of the particles was achieved via tangential flow filtration. The unwashed Ag-nps solution contained approximately 5.55 mg/mL formaldehyde solvent and methanol by-product from their formation. These unwashed Ag-nps were then ultracentrifuged to obtain the solution supernatant for toxicity testing (“as synthesized” supernatant). The Ag-nps were then serially washed with 20 vol equivalents of 2 mM phosphate buffer (pH 7.5) and the 5, 10, 15, and 20 washing permeates were collected. The washing permeates contained 20–50 ppb dissolved Ag. The colloidal Ag-nps were stored at 4°C in the dark. The carbon-coated Ag-nps, synthesized by pulsed plasma reactor and coated with polyaromatic graphitic carbon, were supplied as a powder and stored at room temperature.

### Cell culture

Cryopreserved primary neonatal HEKs (Lonza, Walkersville, MD) were grown in Keratinocyte Growth Medium-2 (KGM-2; Lonza) in cell culture flasks (75 cm^2^; 1,000,000 cells) to approximately 80% confluency in a 37°C humidified 5% CO_2_ incubator. The cells were passed into clear or black 96-well microplates (12,500 cells/well; 200 μL) in which the peripheral wells contained only KGM-2 to prevent the evaporation of treatment medium. Between 18 and 24 hr later, after reaching approximately 80% confluency, the HEKs were exposed to either KGM-2 (control) or serial dilutions of each Ag-nps type for the following experiments.

### Evaluation of best viability assay

A nanoparticle (no-cell) control (nanoparticles in collagen-coated wells with no HEKs) and a nanoparticle/cell control (nanoparticles exposed to HEK-metabolized assay dye) were run in parallel with each viability assay as described by Monteiro-Riviere et al. (2009) to assess the interactions between the viability assays and the Ag-nps [see Supplemental Material available online (doi:10.1289/ehp.0901398.S1 via http://dx.doi.org/)].

### Ag-nps treatment of HEKs

We conducted an initial dose–response study to assess the concentrations of Ag-nps that could affect HEK after 24-hr exposure. Most of the colloidal Ag-nps tested were supplied in both low volume and concentration, which limited the highest HEK dosing concentration to 1.7 μg/mL. Combined with KGM-2 medium, a 1.7 μg/mL solution of the Ag-nps was serially diluted (1:5) to provide concentrations ranging from 1.7 to 0.000544 μg/mL. The effect of the Ag-nps on HEK viability was assessed by three different toxicity assays: 3-(4,5-dimethylthiazol-2-yl)-2,5-diphenyltetrazolium bromide (MTT; Sigma-Aldrich, St. Louis, MO), alamarBlue (aB; Molecular Probes, Invitrogen, Eugene, OR), and CellTiter 96 AQueous One (96AQ; Promega, Madison, WI) [for details, see Supplemental Material (doi:10.1289/ehp.0901398.S1 via http://dx.doi.org/)].

We tested dosing concentrations of the washed and carbon-coated Ag-nps up to 42.5 μg/mL but saw no response. Additionally, to differentiate the potential cytotoxicity between the particles and the contaminants present in the colloidal solution, HEKs were treated with the “as synthesized” supernatant and the 5, 10, 15, and 20 washing permeate for 24 hr at concentrations ranging from 1.7 μg/mL to 0.068 μg/mL.

### Cytokine release

For the concentrations of Ag-nps that showed toxicity, we conducted cytokine analysis to determine their proinflammatory potential by assessing the release of interleukin (IL)-8, IL-6, tumor necrosis factor-α (TNF-α), IL-10, and IL-1β; the procedure is described in the Supplemental Material (doi:10.1289/ehp.0901398.S1).

### *In vivo* porcine skin exposure

We compared the effects of unwashed Ag-nps with those of washed Ag-nps *in vivo*. Assuming the two smallest Ag-nps could penetrate the skin, the comparison was limited to the 20- and 50-nm washed and unwashed samples. Pigs were dosed on the back skin with Ag-nps solutions ranging from 34.0 to 0.34 μg/mL [for details, see Supplemental Material (doi:10.1289/ehp.0901398.S1)]. Skin was evaluated for erythema and edema according to the Draize system (Draize et al. 1944): for erythema: 0, no change; 1, very slight change; 2, pale red in defined area; 3, definite red in well-defined area; 4, crimson red; and for edema: 0, no change; 1, very slight change; 2, slight change with edges barely defined; 3, moderate change, with area raised 1 mm; 4, severe change, with area raised > 1 mm and extending beyond the exposure area. All animals were treated humanely and with regard for alleviation of suffering.

### Microscopic observations

To assess morphologic alterations during the *in vivo* study, we harvested tissue samples after the pigs were euthanized and processed them routinely for light microscopy [see Supplemental Material (doi:10.1289/ehp.0901398.S1)]. Approximately 1-cm sections were evaluated for intercellular and intracellular epidermal edema, dermal edema, and inflammation using the following scoring system: 0, no change; slight, inflammation on less than half the sample; moderate, inflammation on half the sample; severe, inflammation on more than half the sample.

### Ultrastructural observations

Particle size analysis was conducted with both DLS and TEM to confirm the manufacturer-identified diameters and surface characterization [for details, see Supplemental Material (doi:10.1289/ehp.0901398.S1)]. To localize Ag-nps uptake *in vitro*, HEKs were grown to approximately 70% confluency in cell culture flasks (25 cm^2^) and treated for 24 hr with each Ag particle at 1.7 μg/mL in KGM-2. The cells and skin samples were processed routinely for TEM (for details, see Supplemental Material). We viewed all TEM samples on an FEI/Philips EM 208S TEM (FEI, Hillsboro, OR), operating at an accelerating voltage of 80 kV. Additionally, unstained samples were analyzed by X-ray microanalysis [energy dispersive X-ray spectroscopy (EDS)] with a Hitachi HF2000 FE TEM (Hitachi High Technologies America Inc., Lexington, KY) equipped with an Oxford Instruments INCA EDS (Oxford Instruments, Oxfordshire, UK).

### Statistical analysis

We calculated the mean values for HEK percent viability (normalized by viability) and cytokine concentration for each treatment and determined the significant differences (*p* < 0.05) using the PROC GLM procedure (SAS, version 9.1 for Windows; SAS Institute Inc., Cary, NC). When significant differences were found, we performed multiple comparisons using the Tukey’s studentized range highest significant difference test, with *p* < 0.05 as the level of significance. Dunnett’s *t*-test was performed to determine the significance at *p* < 0.5 of differences between control and treatment groups. Data are expressed as the mean ± SEM of two plates (*n* = 6/plate).

## Results

### Evaluation of viability assays

**Figure 1:**
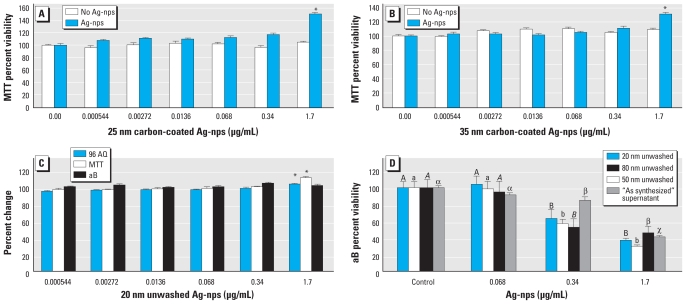
Evaluation of Ag-nps and HEK viability after 24 hr exposure to Ag-nps. Nanoparticle/cell control of HEKs before and after treatment with 25-nm (*A*) or 35-nm carbon-coated Ag-nps (*B*) assessed by MTT. (*C*) Nanoparticle controls (no cells) of 20 nm unwashed Ag-nps assessed by 96 AQ, MTT, and aB. (*D*) HEK viability after 24 hr exposure to various Ag-nps as assessed by aB. *p < 0.05; nanoparticle/cell controls were compared for each concentration before and after Ag-nps, nanoparticle controls were run with multiple comparisons between concentrations, and each Ag-nps type was assessed independently. In *D*, different letters denote significant differences for each nanoparticle at each concentration (*p* < 0.05); each Ag-nps type was assessed independently.

Table 2 summarizes the control studies for the three viability assays, listing the absorbance or fluorescence value for each Ag-nps type that shows a significant interaction. For MTT, at 1.7 μg/mL both the 25-nm (Figure 1A) and 35-nm (Figure 1B) carbon-coated Ag-nps nanoparticle/cell controls show a statistically significant increase in absorbance after exposure of Ag-nps to cell-reacted assay dye. The nanoparticle (no cell) controls showed a statistically significant increase in absorbance at 1.7 μg/mL with the 20-nm unwashed Ag-nps for 96AQ and MTT but not for aB (Figure 1C).

All other nanoparticle control data are available in the Supplemental Material (doi:10.1289/ehp.0901398.S1). We saw no significant changes in the fluorescence values for aB for the 20-, 50-, or 80-nm unwashed Ag-nps (see Supplemental Material, Figure 1a). The unwashed 50- and 80-nm Ag-nps caused a significant increase in absorbance values for MTT and 96AQ but not for aB (see Supplemental Material, Figure 1b,c). The fluorescence value for aB increased significantly at 0.034 μg/mL for both 20- and 50-nm washed Ag-nps and at 1.7 for 80-nm washed Ag-nps (see Supplemental Material, Figure 2a). The absorbance value for 96AQ changed significantly at 1.7 μg/mL for each of the 20-, 50-, and 80-nm washed Ag-nps (see Supplemental Material, Figure 2b). The 80-nm washed Ag-nps caused a significant increase in absorbance value for MTT at 1.7 μg/mL (see Supplemental Material, Figure 2c). The fluorescence value for aB increased significantly at 1.7 μg/mL for 35-nm carbon-coated Ag-nps (see Supplemental Material, Figure 3a). The absorbance value increased significantly for 96AQ at 1.7 μg/mL for 25-nm carbon-coated Ag-nps and at 0.034 μg/mL for 35-nm carbon-coated Ag-nps (see Supplemental Material, Figure 3b). We observed a significant increase in the absorbance value for MTT at 1.7 μg/mL for 25- and 35-nm carbon-coated Ag-nps (see Supplemental Material, Figure 3c).

### Ag-nps treatment of HEK

Generally, for concentrations ranging from 0.000544 to 1.7 μg/mL and exposures of 24 hr, treatment with unwashed Ag-nps resulted in a dose-dependent decrease in viability with all three assays, whereas for the washed and carbon-coated Ag-nps there was no decrease in viability for any assay.

As shown in Figure 1D, the 20- and 50-nm unwashed Ag-nps caused a significant decrease in viability at 0.34 μg/mL for both aB and 96AQ and at 1.7 μg/mL for MTT. The 80-nm unwashed Ag-nps caused a significant decrease in viability at 0.34 μg/mL for all three assays. The “as synthesized” supernatant showed a significant decrease in viability at 0.34 μg/mL for the MTT and aB assays and at 1.7 μg/mL for 96AQ. Toxicity was not present for any of the supernatants obtained from the 5, 10, 15, or 20 washing permeates. HEKs exposed for 24 hr to washed 20-, 50-, or 80-nm Ag-nps or the carbon-coated Ag-nps ranging in concentration from 0.000544 to 1.7 μg/mL showed no significant decrease in viability with any assay [see Supplemental Material, Figures 4–6 (doi:10.1289/ehp.0901398.S1)].

### Cytokine release

**Figure 2:**
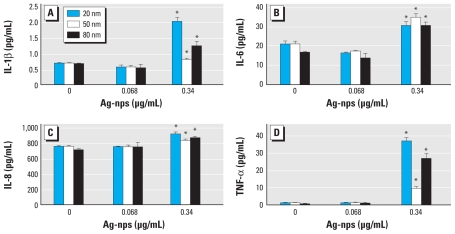
Cytokine release by HEKs treated with 20-, 50-, or 80-nm unwashed Ag-nps. (*A*) IL-1β. (*B*) IL-6. (*C*) IL-8. (*D*) TNF-α. *p < 0.05, by multiple comparisons between concentrations, with each Ag-nps type assessed independently.

We observed a significant increase above the limit of detection in IL-1β (Figure 2A), IL-6 (Figure 2B), IL-8 (Figure 2C), and TNF-α (Figure 2D) from HEKs exposed to 0.34 μg/mL unwashed 20-, 50-, and 80-nm Ag-nps for 24 hr. The lowest limit of detection for IL-1β was 0.8 pg/mL; IL-6, 1.1 pg/mL; IL-8, 0.5 pg/mL; and TNF-α, 3.0 pg/mL. The values for IL-10 were less than the detectable limit of the assay (0.9 pg/mL).

### Macroscopic observations of topically applied Ag-nps on porcine skin

We noted no gross erythema or edema based on the Draize system in any of the treated sites during the entire 14-day *in vivo* study. Treated sites were gray in appearance, representing residual Ag-nps on the surface of the skin.

### Microscopic observations

**Figure 3:**
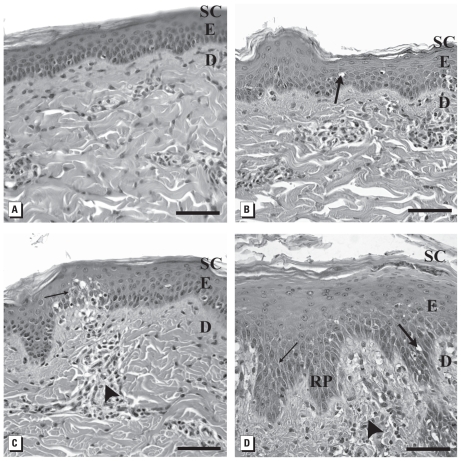
Light micrographs showing effects of 20-nm washed Ag-nps on porcine skin (stained with hematoxylin and eosin). (*A*) Untreated control. Skin exposed to 0.34 μg/mL (*B*); 3.4 μg/mL (*C*); and 34 μg/mL (*D*) 20-nm washed Ag-nps. Abbreviations: D, dermis; E, epidermis; RP, rete peg; SC, stratum corneum. Large arrows indicate intracellular epidermal edema; small arrows, focal areas of intercellular epidermal edema; arrowheads, perivascular inflammation. Bars = 60 μm.

Morphologic observations of untreated porcine skin exhibited normal epidermis and dermis (Figure 3A). Skin dosed daily with Ag-nps for 14 days exhibited a concentration-dependent response, regardless of particle size or if washed or unwashed. Skin treated with the lowest dosing concentration of 0.34 μg/mL 20-nm washed Ag-nps typically showed a slight intracellular and intercellular epidermal edema (Figure 3B); with 3.4 μg/mL of 20-nm washed Ag-nps there was moderate focal intracellular and intercellular epidermal edema and focal epidermal and dermal inflammation (Figure 3C). Skin treated with the highest concentration of 20-nm washed Ag-nps (34 μg/mL) showed severe intracellular and intercellular epidermal edema with severe focal dermal inflammation (spongiosis), epidermal hyperplasia, and parakeratosis. Also, the extension of the rete pegs increased into the superficial papillary layer of the dermis (Figure 3D).

Results for the 20-nm unwashed Ag-nps [see Supplemental Material, Figure 7 (doi:10.1289/ehp.0901398.S1)] were similar to those for the 20-nm washed Ag-nps. In skin treated with 0.34 μg/mL of 20-nm unwashed Ag-nps, we observed slight intracellular epidermal edema; with 3.4 μg/mL, moderate intracellular and intercellular epidermal edema; and with 34 μg/mL, severe intracellular and intercellular epidermal edema with focal areas of intraepidermal infiltrates and superficial papillary dermal inflammation.

### Ultrastructural observations of HEKs exposed to Ag-nps

**Figure 4:**
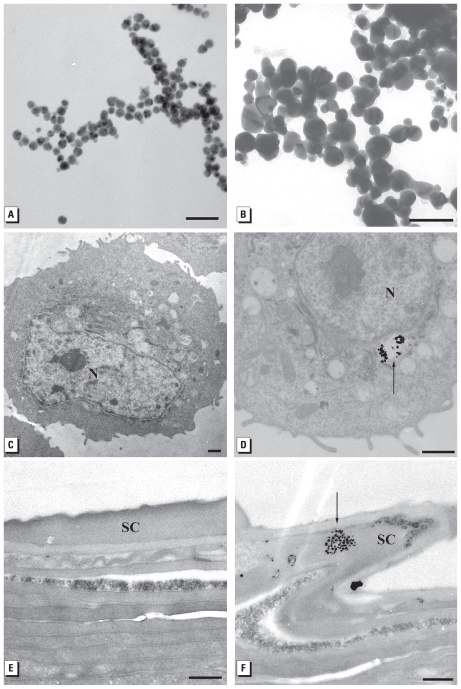
TEM images of (*A*) 20-nm unwashed Ag-nps, (*B*) 25-nm carbon-coated Ag-nps, (*C*) control HEKs, (*D*) 80-nm washed Ag-nps, (*E*) control porcine skin, and (*F*) 34 μg/mL 50-nm washed Ag-nps in stratum corneum of porcine skin. Abbreviations: N, nucleus; unstained sections; SC, stratum corneum. Arrows indicate Ag-nps. Bars = 100 nm for *A* and *B*; 1 μm for *C* and *D*; and 500 nm for *E* and *F*.

Ultrastructural observations of the 20-nm washed and 25-nm carbon- coated Ag-nps are shown in Figure 4A and B [for all other Ag-nps, see Supplemental Material, Figure 8 (doi:10.1289/ehp.0901398.S1)]. The HEK controls appeared normal, with prominent nucleus, nucleolus, and mitochondria (Figure 4C). In HEKs dosed with Ag-nps of all sizes and surface conditions, Ag-nps were localized within membrane- bound cytoplasmic vacuoles; Figure 4D shows a representative image of internalized Ag-nps (for all other Ag-nps electron micrographs, see Supplemental Material, Figure 9). EDS analysis of HEKs dosed with the 20-nm washed Ag-nps confirmed the presence of Ag in the vacuoles; copper from the grid and gold from the particle seed were present (see Supplemental Material, Figure 10).

The control skin consisted of a normal compact stratum corneum with approximately 20–30 layers attached by desmosomes (Figure 4E). For skin dosed daily for 14 days, all Ag-nps localization was within or on top of the stratum corneum. TEM images show the presence of Ag-nps within the superficial layers of the stratum corneum of skin dosed with 34 μg/mL 50-nm washed Ag-nps (Figure 4F), and on top of the stratum corneum of skin dosed with 20-nm washed Ag-nps [see Supplemental Material, Figure 9H (doi:10.1289/ehp.0901398.S1)]. EDS analysis conducted in these areas detected Ag, osmium from the postfixation process, and copper from the grid (see Supplemental Material, Figure 10).

## Discussion

Ag-nps have been integrated into hundreds of products that affect the daily lives of millions of people in many countries. Their main use is for disinfection in wound care and in products such as odor-reducing clothing, acne creams, and face masks. Most of these products come into direct contact with skin, the largest organ of the human body, and could serve as a potential route for nanoparticle penetration. Therefore, the relationship of Ag-nps in skin needs to be investigated with particular focus on their irritation potential, toxicity, and penetration into skin and skin cells. In this study we evaluated the cytotoxic potential of Ag-nps of varying sizes and surface conditions in HEK cells, their penetrating capacity into porcine skin after topical repetitive daily dosing for 2 weeks, and the localization of the Ag-nps within HEKs and porcine skin.

The use of several viability assays is important to determine the optimal assay to assess Ag-nps toxicity; therefore, mortality of HEKs after Ag-nps exposure was evaluated with three different assays that use colorimetric or fluorescent dyes as markers to determine cell viability by assessing cell metabolism.

Nanomaterials, such as single-walled carbon nanotubes (Zhang et al. 2007), carbon black (Monteiro-Riviere and Inman 2006), fullerenes, and QDs (Monteiro-Riviere et al. 2009), are capable of interfering with dye and dye products in viability assays through the adsorption of cell medium constituents and cytokines. We assessed the potential interactions between assays and Ag-nps using nanoparticle and nanoparticle/cell controls, which showed an increase in absorbance and fluorescence values at the highest concentration. The increase in absorbance and fluorescence values could cause the toxicity of the Ag-nps in HEKs to be underestimated. Additionally, the nanoparticle/cell control showed that both the 25- and 35-nm carbon-coated Ag-nps interfered with the MTT assay at the 1.7 μg/mL concentration because of the increase in absorbance values after incubation of the reduced formazan product with Ag-nps (Figure 1A,B). Overall, all assays were affected by Ag-nps; based on its fluorescence values, aB may be the best viability assay to use when conducting experiments with Ag-nps.

MTT, aB, and 96AQ viability assays did not show toxicity for the 25- and 35-nm carbon-coated Ag-nps or for the 20-, 50-, or 80-nm washed Ag-nps. All three assays also showed that the 20-, 50-, and 80-nm unwashed Ag-nps contributed to a decrease in HEK viability 24 hr after exposure to the 0.34–1.7 μg/mL concentrations, but there was no size-dependent decrease in viability. However, the difference in toxicity between the unwashed and washed Ag-nps is inferred to be due to the presence of contaminants in the unwashed solution such as formaldehyde, which has shown to have cytotoxic effects on cell culture (Ku and Billings 1984). In the present study, these residual contaminants were removed by the fifth washing step, as indicated by the lack of cell death after exposure to the 5, 10, 15, and 20 washing supernatants. We also found that Ag-nps of different sizes, surface conditions, and synthesis methods are all internalized into membrane-bound vacuoles in HEKs, without a decrease in viability, after 24 hr.

*In vitro* cell line studies have shown that 25 μg/mL of 25-nm Ag-nps in murine neuroblastoma cells decreases mitochondrial function and causes the production of reactive oxygen species that could potentially contribute to neurodegenerative diseases (Schrand et al. 2008). A significant decrease in mitochondrial function has been shown in hepatic cells after single exposures to 15- and 100-nm Ag-nps at concentrations ranging from 5 to 50 μg/mL (Hussain et al. 2005), in germline stem cells exposed to 10 μg/mL of 15-nm Ag-nps (Braydich-Stolle et al. 2005), and in HEKs and fibroblasts after exposure to approximately 15 μg/mL Ag-nps extracted from commercially available Ag-based wound dressings with Ag content ranging from 13 to 934 μg/cm^2^ (Burd et al. 2007). Interactions between Ag-nps ranging in size from 7 to 20 nm and human skin carcinoma cells showed a decrease in mitochondrial function and the onset of apoptosis at concentrations of 0.78 μg/mL and 1.56 μg/mL, respectively (Arora et al. 2008). The toxic concentrations of the 20-, 50-, and 80-nm unwashed Ag-nps (0.34–1.7 μg/mL) are slightly more sensitive in HEKs compared with *in vitro* toxicity studies conducted by others in different cell lines, although it is important to consider such factors as agglomeration, surface conditions, size, cell lines, and interactions with the assay dye products when comparing across studies.

Keratinocytes produce proinflammatory cytokines, such as IL-8, IL-6, TNF-α, and IL-1β, that serve as mediators for inflammatory and immunologic reactions in skin exposed to irritants (Allen et al. 2000, 2001a, 2001b; Barker et al. 1991; Corsini and Galli 2000; Grone 2002; Monteiro-Riviere et al. 2003; Nickoloff et al. 1991). Although different toxicants may elicit different responses in HEKs, studies in our laboratory have shown cytokine release by HEKs in response to jet fuel exposure (Allen et al. 2000, 2001a, 2001b; Chou et al. 2002, 2003; Monteiro-Riviere et al. 2003, 2004), multiwalled carbon nanotubes (Monteiro-Riviere et al. 2005), 6-aminohexanoic acid-functionalized single-walled carbon nanotubes (Zhang et al. 2007), fullerenes (Rouse et al. 2007), and QDs (Ryman-Rasmussen et al. 2006; Zhang et al. 2008). The inflammatory potential of Ag-nps was confirmed by the increases in IL-1β, IL-6, IL-8, and TNF-α detected in the media from HEK cell cultures exposed to 0.34 μg/mL of each of the unwashed Ag-nps.

Nanomaterials are also capable of being internalized into cells and penetrating through skin; QD-621 have the ability to penetrate into the intercellular lipid layers of the stratum corneum of porcine skin (Zhang et al. 2008); QD-565 and QD-655, with diverse physiochemical properties, have been shown to penetrate into the dermis of abraded skin (Zhang and Monteiro-Riviere 2008); and derivatized fullerenes are localized within the intercellular space of the stratum granulosum layer of flexed excised porcine skin (Rouse et al. 2006). Topical application of 26–30 nm zinc oxide in a sunscreen formulation on *in vitro* human skin localized nanoparticles in the upper stratum corneum with minimal penetration (Cross et al. 2007), and microfine zinc oxide, with a mean size of 80 nm, and agglomerates of titanium dioxide < 160 nm did not penetrate the porcine stratum corneum layer of *in vitro* static diffusion cells (Gamer et al. 2006).

Porcine skin is an excellent model for studying penetration of human skin because its thickness and absorption rates are comparable to those of human skin (Bronaugh et al. 1982; Monteiro-Riviere and Riviere 1996; Reifenrath et al. 1984). In the present study, we were surprised that after 14 consecutive days of topical dosing, the Ag-nps did not cause any macroscopic irritation, although the gray appearance of the skin due to the deposition of Ag-nps may have masked any subtle signs of erythema. When viewed microscopically, focal inflammation and edema increased with an increase in Ag-nps concentration. The highest concentration consistently caused epidermal hyperplasia with elongated extension of rete pegs down into the dermis, which is typical of a chronic irritation reaction as reported with exposure to jet fuels (Monteiro-Riviere et al. 2001; Muhammad et al. 2005). TEM depicted the localization of Ag-nps only in the superficial layers of the stratum corneum, which was similar to results found in a static cell diffusion study (Larese et al. 2009), and suggests that ionic flux into the epidermis could attribute to focal inflammation. Many Ag-nps that were not bound to the skin were washed away during both the light and electron microscopy processing techniques, yet their location is confirmed with other nanoparticles that were not shown to penetrate into the deeper epidermis (Cross et al. 2007; Zhang and Monteiro-Riviere 2008).

## Conclusion

This study indicates that toxicity of Ag-nps in HEKs is influenced by the residual contaminants in the Ag-nps solutions, and that the Ag-nps themselves may not be responsible for an increase in cell mortality. Complete characterization of not only the nanoparticles but also the vehicle is important in order to distinguish between Ag-nps and contaminant toxicity. Additionally, this study shows that 20-, 50-, and 80-nm washed and unwashed Ag-nps, as well as 25- and 35-nm carbon-coated Ag-nps, interfered and/or reacted with MTT, 96AQ, and aB viability assays and that aB may be the best viability assay because of its lower interference with these Ag-nps. Because Ag-nps of several types have been shown to enter cells and remain on the skin, the possibility of Ag-nps entry into the body through damaged or abraded skin is important to consider, particularly because many Ag-containing products are specialized for wound care. With the ability for Ag-nps to enter HEKs, their degradation within the cell may create reactive oxygen species that would be damaging to cell machinery and DNA (Arora et al. 2008). We observed focal inflammation, specifically intracellular and intercellular epidermal edema, after 14 days of topical application of Ag-nps to skin. Studies that use longer exposures (several weeks) and Ag-nps in different vehicles should be carried out, possibly also investigating effects on compromised skin. Overall, the present study provides knowledge onAg-nps toxicity and penetration *in vitro* and *in vivo* over 14 days and provides a basis for occupational and risk assessment.

## Figures and Tables

**Table 1:** Ag-nps properties.

Description	MDD (nm)	DLS diameter (nm)^a^	TEM diameter (nm)^a^	Supplied concentration (mg/mL)	Particle concentration (particles/mL)^a^	Zeta^b^ potential (mV)
Unwashed, colloid	20	30.8 ± 0.6	22.4 ± 2.6	0.20	2.41 × 10^12^	−29.7
	50	47.7 ± 0.5	49.4 ± 6.2	0.20	4.44 × 10^11^	−27.8
	80	75.5 ± 1.0	79.2 ± 8.0	0.20	7.09 × 10^10^	−33.2
Washed, colloid	20	25.5 ± 0.4	21.4 ± 3.1	2.86	1.89 × 10^14^	−46.0
	50	43.7 ± 1.1	50.0 ± 5.9	3.45	5.01 × 10^12^	−44.3
	80	79.9 ± 28.0	77.0 ± 6.0	2.79	1.07 × 10^12^	−43.7
Carbon-coated, powder	25	149.0 ± 89	27.2 ± 10.3	NA	NA	−24.0
	35	167.0 ± 110	37.0 ± 11.6	NA	NA	−29.0

Abbreviations: MDD, manufacturer-designated diameter; NA, not applicable.

a Mean ± SD.

b Zeta potential in deionized water.

**Table 2:** Significant change in absorbance or fluorescence values (μg/ml Ag-nps/mL media) in control studies by assay.

	No cell control^a^			Cell control^b^		
Ag-nps	MTT	aB	96AQ	MTT	aB	96AQ
Unwashed						
20 nm	1.7	[Table-fn tfn4-ehp-118-407]—	1.7	[Table-fn tfn4-ehp-118-407]—	[Table-fn tfn4-ehp-118-407]—	[Table-fn tfn4-ehp-118-407]—
50 nm	0.34	[Table-fn tfn4-ehp-118-407]—	1.7	[Table-fn tfn4-ehp-118-407]—	[Table-fn tfn4-ehp-118-407]—	[Table-fn tfn4-ehp-118-407]—
80 nm	1.7	[Table-fn tfn4-ehp-118-407]—	1.7	[Table-fn tfn4-ehp-118-407]—	[Table-fn tfn4-ehp-118-407]—	[Table-fn tfn4-ehp-118-407]—

Washed						
20 nm	[Table-fn tfn4-ehp-118-407]—	0.34	1.7^c^	[Table-fn tfn4-ehp-118-407]—	[Table-fn tfn4-ehp-118-407]—	[Table-fn tfn4-ehp-118-407]—
50 nm	[Table-fn tfn4-ehp-118-407]—	1.7	1.7	[Table-fn tfn4-ehp-118-407]—	[Table-fn tfn4-ehp-118-407]—	[Table-fn tfn4-ehp-118-407]—
80 nm	1.7	1.7	1.7	[Table-fn tfn4-ehp-118-407]—	[Table-fn tfn4-ehp-118-407]—	[Table-fn tfn4-ehp-118-407]—

Carbon-coated						
25 nm	1.7	[Table-fn tfn4-ehp-118-407]—	1.7	1.7	[Table-fn tfn4-ehp-118-407]—	[Table-fn tfn4-ehp-118-407]—
35 nm	1.7	[Table-fn tfn4-ehp-118-407]—	0.34	1.7	[Table-fn tfn4-ehp-118-407]—	[Table-fn tfn4-ehp-118-407]—

— No change in absorbance or fluorescence value was found, indicating a lack of interaction between assay and Ag-nps.

a No cell control value at which a significant increase in absorbance or fluorescence was observed (*p* < 0.05).

b Cell control value at which a significant increase in absorbance was observed before and after Ag-nps (*p* < 0.05).

c A significant decrease was observed for this data set.

## References

[b1-ehp-118-407] Allen DG, Riviere JE, Monteiro-Riviere NA (2000). Identification of early biomarkers of inflammation produced by keratinocytes exposed to jet fuels jet A, JP-8, and JP-8(100). J Biochem Mol Toxicol.

[b2-ehp-118-407] Allen DG, Riviere JE, Monteiro-Riviere NA (2001a). Analysis of interleukin-8 release from normal human epidermal keratinocytes exposed to aliphatic hydrocarbons: delivery of hydrocarbons to cell cultures via complexation with alpha-cyclodextrin. Toxicol In Vitro.

[b3-ehp-118-407] Allen DG, Riviere JE, Monteiro-Riviere NA (2001b). Cytokine induction as a measure of cutaneous toxicity in primary and immortalized porcine keratinocytes exposed to jet fuels, and their relationship to normal human epidermal keratinocytes. Toxicol Lett.

[b4-ehp-118-407] Arora S, Jain J, Rajwade JM, Paknikar KM (2008). Cellular responses induced by silver nanoparticles: in vitro studies. Toxicol Lett.

[b5-ehp-118-407] Barker JN, Jones ML, Swenson CL, Sarma V, Mitra RS, Ward PA (1991). Monocyte chemotaxis and activating factor production by keratinocytes in response to IFN-gamma. J Immunol.

[b6-ehp-118-407] Benn TM, Westerhoff P (2008). Nanaoparticle silver released into water from commercially available sock fabrics. Environ Sci Technol.

[b7-ehp-118-407] Braydich-Stolle L, Hussain S, Schlager J, Hofmann MC (2005). *In vitro* cytotoxicity of nanoparticles in mammalian germ line stem cells. Toxicol Sci.

[b8-ehp-118-407] Bronaugh RL, Stewart RF, Congdon ER (1982). Methods for *in vitro* percutaneous absorption studies II: animal models for human skin. Toxicol Appl Pharmacol.

[b9-ehp-118-407] Burd A, Kwok CH, Hung SC, Chan HS, Gu H, Lam WK (2007). A comparative study of the cytotoxicity of silver-based dressings in monolayer cell, tissue explant, and animal models. Wound Repair Regen.

[b10-ehp-118-407] Chou CC, Riviere JE, Monteiro-Rivere NA (2002). Differential relationship between the carbon chain length of jet fuel aliphatic hydrocarbons and their ability to induce cytotoxicity vs. interleukin-8 release in human epidermal keratinocyties. Toxicol Sci.

[b11-ehp-118-407] Chou CC, Riviere JE, Monteiro-Riviere NA (2003). The cytotoxicity of jet fuel aromatic hydrocarbons and dose-related interleukin-8 release from human epidermal keratinocytes. Arch Toxicol.

[b12-ehp-118-407] Corsini E, Galli CL (2000). Epidermal cytokines in experimental contact dermatitis. Toxicology.

[b13-ehp-118-407] Crede CSF (1881). Die Verhutung der Augenentzundung der Neugeborenen. Arch Gynakol.

[b14-ehp-118-407] Cross SE, Brian I, Roberts MS (2007). Human skin penetration of sunscreen nanoparticles: *in vitro* assessment of a novel micronized zinc oxide formulation. Skin Pharmacol Physiol.

[b15-ehp-118-407] Danscher G (1980). Light and electron microscopic localization of silver in biological tissue. Histochem Cell Biol.

[b16-ehp-118-407] Draize JH, Woodard G, Calvery HO (1944). Methods for the study of irritation and toxicity of substances applied to the skin and mucous membranes. J Pharmacol Exp Ther.

[b17-ehp-118-407] Fung MC, Bowen DL (1996). Silver products for medical indications: risk-benefit assessment. Clin Toxicol.

[b18-ehp-118-407] Gamer AO, Leibold E, van Ravenzwaay B (2006). The *in vitro* absorption of microfine zinc oxide and titanium dioxide through porcine skin. Toxicol In Vitro.

[b19-ehp-118-407] Gong P, Li H, He X, Wang K, Hu J, Tan W (2007). Preparation and antibacterial activity of Fe_3_O_4_–Ag nanoparticles. Nanotechnology.

[b20-ehp-118-407] Grone A (2002). Keratinocytes and cytokines. Vet Immunol Immunopathol.

[b21-ehp-118-407] Hussain S, Hess K, Gearhart J, Geiss K, Schlager J (2005). *In vitro* toxicity of nanoparticles in BRL3A rat liver cells. Toxicol In Vitro.

[b22-ehp-118-407] Ku RH, Billings RE (1984). Relationships between formaldehyde metabolism and toxicity and glutathione concentrations in isolated rat hepatocytes. Chem Biol Interact.

[b23-ehp-118-407] Larese FF, D’Agostin F, Crosera M, Adami G, Renzi N, Bovenzi M (2009). Human skin penetration of silver nanoparticles through intact and damaged skin. Toxicology.

[b24-ehp-118-407] Lee SM, Lee SH (1994). Generalized argyria after habitual use of silver nitrate. J Dermatol.

[b25-ehp-118-407] Marshall JP, Schneider RP (1977). Systemic argyria secondary to topical silver nitrate. Arch Dermatol.

[b26-ehp-118-407] Monteiro-Riviere N, Inman A, Riviere J (2001). The effects of short-term high-dose and low-dose dermal exposure to Jet A, JP-8, and JP-8+100 jet fuels. J Appl Toxicol.

[b27-ehp-118-407] Monteiro-Riviere NA, Baynes RE, Riviere JE (2003). Pyridostigmine bromide modulates topical irritant-induced cytokine release from human epidermal keratinocytes and isolated perfused porcine skin. Toxicology.

[b28-ehp-118-407] Monteiro-Riviere NA, Inman AO (2006). Challenges for assessing carbon nanomaterial toxicity to the skin. Carbon.

[b29-ehp-118-407] Monteiro-Riviere NA, Inman AO, Riviere JE (2004). Skin toxicity of jet fuels: ultrastructural studies and the effects of substance P. Toxicol Appl Pharmacol.

[b30-ehp-118-407] Monteiro-Riviere NA, Inman AO, Zhang LW (2009). Limitations and relative utility of screening assays to assess engineered nanoparticle toxicity in a human cell line. Toxicol Appl Pharmacol.

[b31-ehp-118-407] Monteiro-Riviere NA, Nemanich RJ, Inman AO, Wang YY, Riviere JE (2005). Multi-walled carbon nanotube interactions with human epidermal keratinocytes. Toxicol Lett.

[b32-ehp-118-407] Monteiro-Riviere NA, Riviere JE, Tumbleson ME, Schook LB (1996). The pig as a model for cutaneous pharmacology and toxicology research. Advances in Swine in Biomedical Research.

[b33-ehp-118-407] Moyer CA, Brentano L, Gravens DL, Margraf HW, Monafo WW (1965). Treatment of large human burns with 0.5% silver nitrate solution. Arch Surg.

[b34-ehp-118-407] Muhammad F, Monteiro-Riviere NA, Riviere JE (2005). Comparative in vivo toxicity of topical JP-8 jet fuel and its individual hydrocarbon components: identification of tridecane and tetradecane as key constituents responsible for dermal irritation. Toxicol Pathol.

[b35-ehp-118-407] National Center for Complementary and Alternative Medicine (2009). Colloidal Silver Products. Publication no. D209.

[b36-ehp-118-407] National Institute for Occupational Safety and Health (2003). Registry of Toxic Effects of Chemical Substances: Silver. RTECS #: VW3500000. CAS #: 7440-22-4.

[b37-ehp-118-407] Nickoloff BJ, Varani J, Mitra RS (1991). Modulation of keratinocyte biology by gamma interferon: relevance to cutaneous wound healing. Prog Clin Biol Res.

[b38-ehp-118-407] Project on Emerging Nanotechnologies (2009). Analysis.

[b39-ehp-118-407] Reifenrath WG, Chellquist EM, Shipwash EA, Jederberg WW (1984). Evaluation of animal models for predicting skin penetration in man. Fundam Appl Toxicol.

[b40-ehp-118-407] Rouse JG, Yang J, Barron AR, Monteiro-Riviere NA (2007). Fullerene-based amino acid nanoparticle interactions with human epidermal keratinocytes. Toxicol In Vitro.

[b41-ehp-118-407] Rouse JG, Yang J, Ryman-Rasmussen J, Barron AR, Monteiro-Riviere NA (2006). Effects of mechanical flexion on the penetration of fullerene amino acid-derivatized peptide nanoparticles through skin. Nano Lett.

[b42-ehp-118-407] Rungby J, Danscher G (1983). Localization of exogenous silver in brain and spinal cord of silver exposed rats. Acta Neuropathol.

[b43-ehp-118-407] Ryman-Rasmussen J, Riviere JE, Monteiro-Riviere NA (2006). Penetration of intact skin by quantum dots with diverse physicochemical properties. Toxicol Sci.

[b44-ehp-118-407] Schrand AM, Braydich-Stolle LK, Schlager JJ, Dai L, Hussain SM (2008). Can silver nanoparticles be useful as potential biological labels?. Nanotechnology.

[b45-ehp-118-407] Temple RM, Farooqi AA (1985). An elderly, slate-grey woman. Practitioner.

[b46-ehp-118-407] U.S. Environmental Protection Agency (2003). Silver (CASRN 7440-22-4).

[b47-ehp-118-407] Zhang LW, Monteiro-Riviere NA (2008). Assessment of quantum dot penetration into intact, tape-stripped, abraded and flexed rat skin. Skin Pharmacol Physiol.

[b48-ehp-118-407] Zhang LW, Yu WW, Colvin VL, Monteiro-Riviere NA (2008). Biological interactions of quantum dot nanoparticles in skin and in human epidermal keratinocytes. Toxicol Appl Pharmacol.

[b49-ehp-118-407] Zhang LW, Zeng L, Barron AR, Monteiro-Riviere NA (2007). Biological interactions of functionalized single-wall carbon nanotubes in human epidermal keratinocytes. Int J Toxicol.

